# Kinetic characterization of amino acid activation by aminoacyl‐tRNA synthetases using radiolabelled γ‐[^32^ P]ATP

**DOI:** 10.1002/2211-5463.13903

**Published:** 2024-09-30

**Authors:** Igor Živković, Morana Dulic, Petra Kozulic, Marko Mocibob, Ita Gruic‐Sovulj

**Affiliations:** ^1^ Department of Chemistry, Faculty of Science University of Zagreb Zagreb Croatia

**Keywords:** amino acid activation, aminoacyl‐tRNA synthetases, ATP/PP_i_ exchange, isotopic equilibrium exchange

## Abstract

Aminoacyl‐tRNA synthetases (AARSs) are fundamental enzymes that pair amino acids and tRNAs for protein synthesis. Aminoacylation occurs in two discrete steps. The amino acid is first activated by ATP, leading to an aminoacyl‐adenylate intermediate and pyrophosphate (PP_i_) formation. In a subsequent step, the aminoacyl moiety is transferred to the tRNA. Kinetic assays were developed to follow each of these steps independently, as well as cumulative two‐step aminoacylation. The main advantage of following the activation step over two‐step aminoacylation is that most AARSs can activate amino acids in the absence of the tRNA, the production of which is laborious. Hence, the activation step is often tested first in the kinetic analysis, including large screens exploring AARS‐targeting inhibitors. Since the 1960s, the activation reaction has been routinely followed by the standard ATP/[^32^P]PP_i_ exchange assay, which relies on the equilibrium exchange of radiolabel between PP_i_ and ATP using [^32^P]PP_i_ as a labelled compound. However, this method became much less convenient when [^32^P]PP_i_ was discontinued in 2022. As a solution, we developed a modified assay that uses easily attainable γ‐[^32^P]ATP as a labelled compound in the equilibrium‐based assay. Using this assay, herein named the [^32^P]ATP/PP_i_ assay, we followed the activation step of several AARSs. The obtained data are in good agreement with the previously published kinetic constants obtained with the standard ATP/[^32^P]PP_i_ exchange assay.

## Graphical abstract

Activation of amino acids by aminoacyl‐tRNA synthetases can be followed by a modified ATP/PP_i_ exchange assay using radiolabelled γ‐[^32^P]ATP. The reaction is quenched at certain time points, followed by separation of the product ([^32^P]PP_i_) from the substrate (γ‐[^32^P]ATP) by thin‐layer chromatography (TLC). Phosphor storage screens are exposed to TLC plates, and results visualized with a Typhoon biomolecular imager.

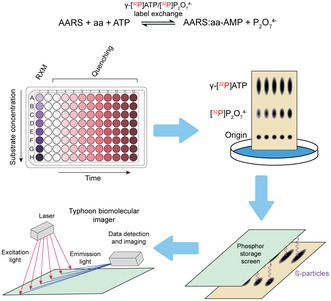

**Abbreviations**
AARS: aminoacyl‐tRNA synthetase
AMP: adenosine 5′‐monophosphate
ATP: adenosine 5′‐triphosphate
IleRS: isoleucyl‐tRNA synthetase
LeuRS: leucyl‐tRNA synthetase
PPi: pyrophosphate
TLC: thin layer chromatography

Aminoacyl‐tRNA synthetases (AARSs) are essential enzymes, which decode genetic information by coupling cognate amino acids and tRNAs for ribosomal protein biosynthesis [1, 2]. They are divided into two evolutionary distinct classes, I and II [3]. AARSs from both classes use the same chemistry and catalyse the aminoacylation reaction, which proceeds in two distinct chemical steps at the same active site (Fig. 1) [4]. First, the ATP activates the amino acid to form aminoacyl‐AMP (aa‐AMP) and PP_i_ [5, 6, 7]. In the second step, the aminoacyl moiety is transferred to the tRNA leading to aminoacylated tRNA (aa‐tRNA) [8, 9]. Due to their role in nature, AARSs have been extensively studied for almost three‐quarters of a century. A quick look at the Web of Science database reveals nearly 1500 papers on “aminoacyl tRNA synthetase” just in the last decade, showing an everlasting interest for AARS in the scientific community.

**Fig. 1:**
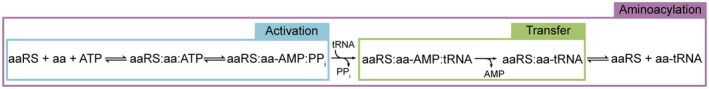
Aminoacyl‐tRNA synthetase aminoacylation pathway. Amino acid is first activated by the ATP (blue box). Activated amino acid is then transferred to the tRNA (green box). The full aminoacylation pathway (purple box) is marked by the dissociation of the aminoacyl‐tRNA from an enzyme.

Several steady‐state and transient kinetic assays have been developed to address AARS complex kinetic mechanisms [10, 11]. Cumulative two‐step aminoacylation is routinely studied using amino acids radiolabelled with ^14^C, ^3^H or ^32^S, or tRNA labelled with ^32^P [10, 11, 12]. Further, some attempts were made towards developing radiolabel‐free methods for testing aminoacylation [13, 14, 15, 16, 17]. Besides the cumulative reaction, each reaction step, amino acid activation, and aminoacyl transfer can be isolated and studied separately [11], providing a full understanding of AARS mechanisms and features. The amino acid activation step can be taken as a proxy of AARS activity and hence used for testing potential antibiotics/inhibitors [18, 19]. Furthermore, measuring the activation step is a critical tool for determining the initial amino acid selectivity of AARS [20, 21, 22]. Most AARS do not need tRNA to activate amino acids, and thus, their activation step can be followed in the absence of tRNA, the production of which can be expensive and/or cumbersome. Exceptions are arginyl‐, glutamyl‐, glutaminyl‐, and class I lysyl‐tRNA synthetase [23, 24, 25, 26], which activate the amino acid in a tRNA‐dependent fashion.

The activation step is routinely measured by ATP/[^32^P]PP_i_ exchange assay, which follows isotopic (^32^P) exchange between PP_i_ and ATP at reaction equilibrium [10, 27, 28]. Except for AARS, ATP/[^32^P]PP_i_ exchange is commonly used for enzymes from the ANL (Acyl‐CoA synthetases, the adenylation domain of the modular nonribosomal peptide synthetases, NRPSs, and Luciferases) superfamily [29, 30, 31]. In this assay, pyrophosphate (PP_i_, P_2_O_7_^4−^) spiked with [^32^P]PP_i_ is added to the reaction mixture containing AARS, ATP, and amino acid. Because equilibrium between unlabelled species is attained instantaneously, a label can be added simultaneously with the unlabelled substrate [28]. The equilibrium rate of [^32^P]ATP formation is taken as a measure of the activation step. It is important to emphasize that the activation cannot be measured by following the formation of aa‐AMP and PP_i_ in a multi‐turnover (steady‐state) assay, because the aa‐AMP (intermediate) dissociation step, which is out of the AARS reaction pathway and thus is very slow, will mask a faster chemical step of the activation. In principle, the chemical step of aa‐AMP formation can be followed directly only by the transient kinetic methods [32], which are more complicated and require special equipment (rapid chemical quench or stopped flow). Recently, an assay following the activation step indirectly through the usage of Ap4A (diadenosine 5′,5‴‐P1,P4‐tetraphosphate) was developed [33, 34]. Although interesting, that assay may not be applicable for all AARSs making the equilibrium isotopic exchange the method of choice for testing the amino acid activation. However, in the summer of 2022, [^32^P]PP_i_ (Revvity, Waltham, MA, USA, ex Perkin‐Elmer, Waltham, MA, USA, cat no. NEX019) was discontinued. Even though [^32^P]PP_i_ could still be ordered as a custom‐synthesized radiochemical, it was far more expensive and far less accessible. This made standard ATP/PP_i_ exchange assay significantly less convenient. To overcome this, we established a protocol that substitutes the radiolabelled [^32^P]PP_i_ for readily available γ‐[^32^P]ATP (cat no. BLU002Z; Revvity, Waltham, MA, USA), which we describe here.

## Materials

### Activation reaction

1. Reaction buffer: HEPES (cat no. BP310‐100; Fisher Scientific), potassium hydroxide (cat no. 1152866; Kemika, Zagreb, Croatia), magnesium chloride hexahydrate (cat no. P139130; Kemika), potassium chloride (cat no. 1160206; Kemika), dithiothreitol (cat no. 6098.3; Roth, Karlsruhe, Germany), bovine serum albumin (cat no. B9200S; New England Biolabs Ipswich, MA, USA).
2. Other reaction components/substrates: sodium pyrophosphate (cat no. P‐8010; Sigma, Burlington, MA, USA), adenosine 5′‐triphosphate disodium salt hydrate (cat no. A2383; Sigma), l‐leucine (cat no. 61819; Sigma‐Aldrich, Burlington, MA, USA), l‐isoleucine (cat no. 58879; Sigma‐Aldrich), γ‐[^32^P]ATP (cat no. BLU002Z; Revvity).
3. Quench solution: sodium acetate (cat no. P143410; GramMol, Zagreb, Croatia), acetic acid (cat no. UN2789; LabExpert, Vilinus, Lithuania), sodium dodecyl sulphate (cat no. 23262; Roth).
4. Eppendorf tubes 1.5 mL or equivalent.
5. Microtiter plates (cat no. G080‐UB; Kisker Biotech, Steinfurt, Germany) and caps (cat no. G080‐D; Kisker Biotech).
6. Dry block heater (cat no. QBD2; Grant, Royston, UK).
7. Multichannel pipette.
8. pH strips (cat no. 92118; Macherey‐Nagel, Düren, Germany)
9. Plexiglass shield.

### Thin layer chromatography

1. Thin‐layer chromatography polyethyleneimine plates (cat no. 801063; Macherey‐Nagel).
2. Glass or plexiglass box for (pre‐)developing plates.
3. Mobile phase/developing buffer: urea (cat no. P167321; GramMol), potassium dihydrogen phosphate (cat no. 60221; Honeywell‐Fluka, Charlotte, NC, USA), phosphoric acid (cat no. 7664‐38‐2; T.T.T., Sveta Nedjelja, Croatia).
4. Magnetic mixer and pH meter.
5. Hair dryer.

### Imaging and analysis

1. Storage Phosphor Screen Image Eraser (cat no. 29‐1871‐90; Cytiva, Marlborough, MA, USA).
2. Phosphor storage screen BAS‐IP MS (cat no. 28‐9564‐74; Cytiva).
3. Exposure cassette (cat no. 63‐0035‐45; Cytiva).
4. Amersham Typhoon biomolecular imager IP (cat no. 29‐1871‐94; Cytiva).
5. Amersham Typhoon Scanner software 2.0.0.6 (Cytiva).
6. imagequant software 8.2.0 (Cytiva).
7. Microsoft Excel.

## Methods

### Activation reaction

1. Standard reaction mixture contains 20–50 mm HEPES‐KOH pH 7.5 (depending on the amino acid concentration and buffering capacity—check the pH of a reaction mixture with a pH strip), 10–20 mm MgCl_2_ (at least equimolar to the ATP concentration), 5 mm DTT, 0.1 mg·mL^−1^ BSA, 1 mm PP_i_, 10–100 nm AARS, 0.1–10 *K*_m_ amino acid and 4 mm ATP (pH 7.5) supplemented with γ‐[^32^P]ATP of activity 0.0001–0.001 mCi·mL^−1^ in total volume of 20 μL.
2. Typically, eight reactions (corresponding to eight different amino acid concentrations – 0.1–10 *K*_m_) are performed on one microplate.
3. Master mix is prepared without the addition of amino acids. The concentration of components in the master mix is two times higher than their final concentrations.
4. 10 μL of the master mix is pipetted in the wells (column 1).
5. The amino acid is pipetted on the same plate, one well for every reaction (column 2). The amino acid concentration in Column 2 is twice the final concentration.
6. Quench solution (600 mm NaOAc pH 4.5 and 0.15% SDS), typically 3 μL, is pipetted on the same plate. Each well is used for a single time point, with typically five to 10 time points aliquoted per reaction mixture across columns 3–12.
7. The plate is left to incubate at a given temperature for a few minutes.
8. Reaction is started by the addition of 10 μL of amino acids (Column 2) to the master mix (Column 1).
9. 1.5 μL aliquots of the reaction mixture are mixed with 3 μL of quench (i.e., quenched) at certain time points.

### Product separation by thin‐layer chromatography

1. Before chromatography, TLC plates are cut into required sizes—10 cm height and 1 cm width per time point. The TLC plates are pre‐activated by being developed with water as a mobile phase.
2. After pre‐activation plates are air dried.
3. Quenched time points are spotted on the TLC plates (app. 1.5 cm above the bottom) and chromatography is performed in the ATP/PP_i_ mobile phase (4 m urea and 750 mm KH_2_PO_4_ pH 3.5).
4. After the mobile phase reaches the top of the plate, it can be left to airdry or dried by a hairdryer.
5. Plates are wrapped in household plastic wrap to prevent cross‐contamination and excess moisture from reaching the phosphor storage screen.
6. Phosphor storage screens are exposed to plates in an exposure cassette.
7. Depending on the specific activity of the γ‐[^32^P]ATP stock (*t*_1/2_ = 14.3 days), plates are left for exposure 2–24 h.

### Imaging and analysis

**Fig. 2:**
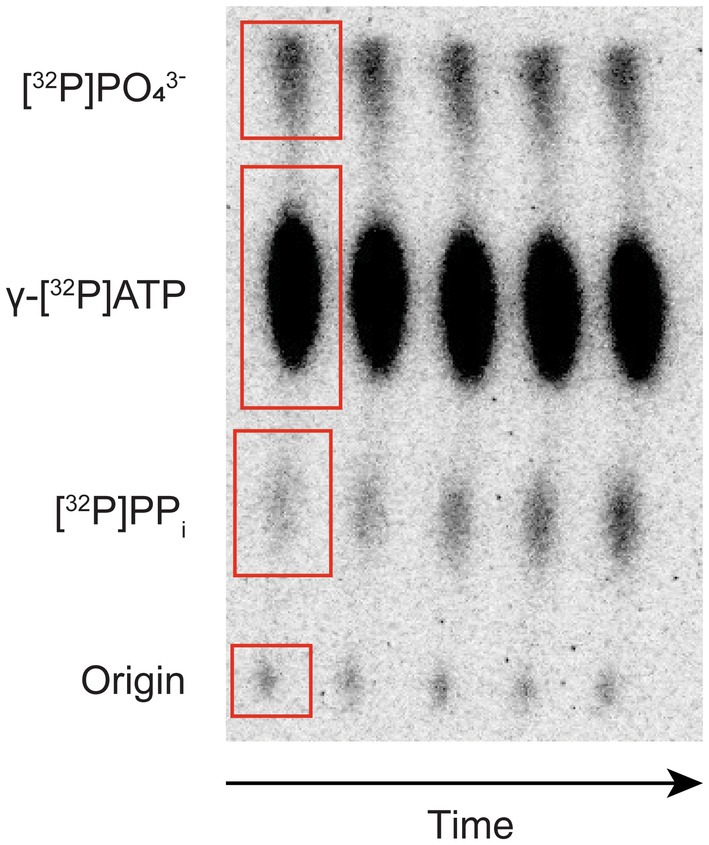
A thin‐layer chromatogram of an amino acid activation time course showing separation of [^32^P]PP_i_ product from the remaining [^32^P]ATP. The other two signals represent the origin and the [^32^P]P_i_ produced by spontaneous hydrolysis of [^32^P]ATP and/or [^32^P]PP_i_. Signals are defined by red rectangles.

**Fig. 3:**
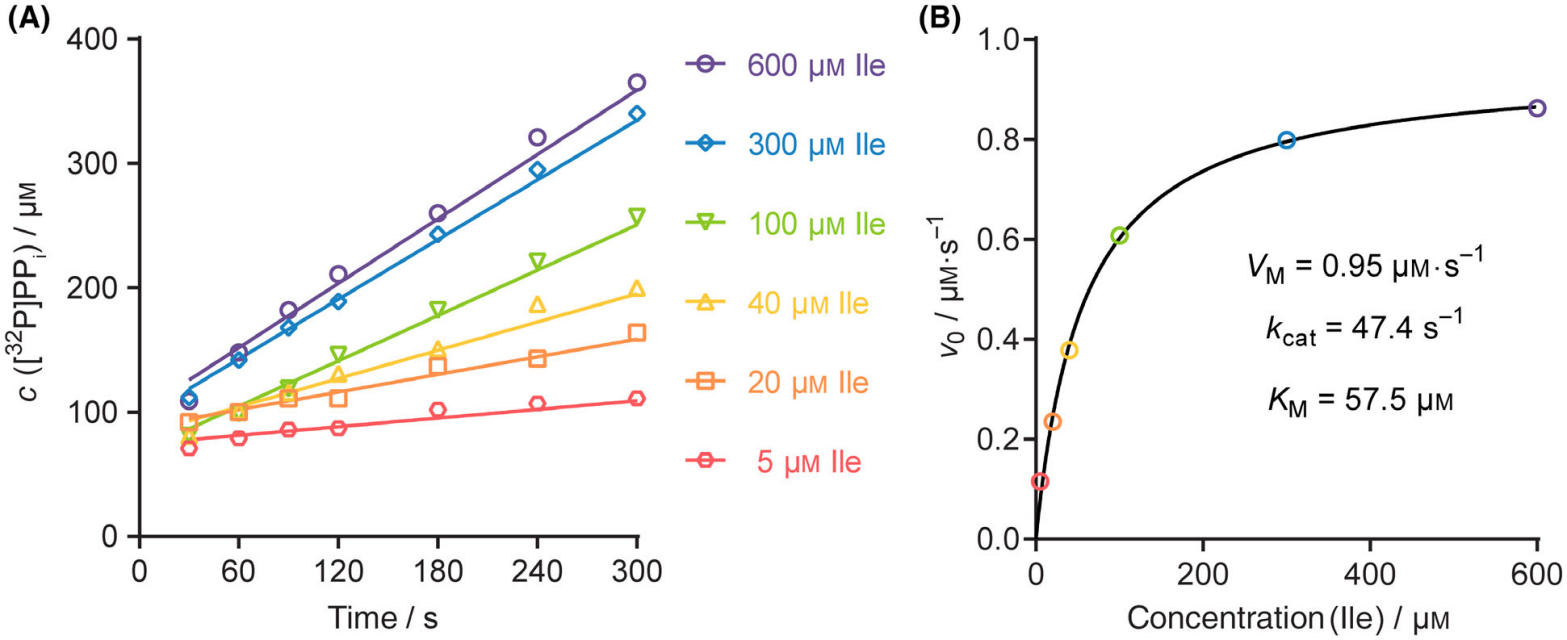
Representative data analysis for the isoleucine activation by *Priestia megaterium* isoleucyl‐tRNA synthetase 2. Data of a single representative experiment (*n* = 1) are shown. The data for three independent (*n* = 3) experiments are given in Table 1. (A) A pyrophosphate concentration plotted against time for different substrate concentrations. (B) Michaelis–Menten fit obtained by plotting the initial velocities against substrate concentration. Michaelis constant and maximum velocity are extracted from the non‐linear regression. The turnover number is calculated by dividing maximal velocity by total enzyme concentration.

1. After exposure, TLC plates are removed from the phosphor storage screens.
2. The screen is visualized on a Typhoon biomolecular imager. Phosphor imaging mode is used with the following setup: Pixel size—200 μm and Sensitivity—4000.
3. A typical time course will generate the image as shown in Fig. 2.
  - Origin always shows low‐intensity signals that can be ignored.
  - As the reaction occurs, pyrophosphate is produced, which is observed by the increase in its signal intensity in time.
  - Simultaneously, the decrease in the γ‐[^32^P]ATP intensity is small due to the initial velocity conditions being followed (i.e., following a short initial period of the reaction).
  - The uppermost signal belongs to the orthophosphate, which is a product of the stock γ‐[^32^P]ATP spontaneous hydrolysis. There should be no observable change in the signal intensity over time, and thus, it can be ignored from calculations (see 5.).
4. Signals are analysed by the imagequant software (8.2.0.). Rectangles are drawn around signals. After all signals have been selected, a background rectangle is drawn in the empty part of the plate. Signals are corrected for the background. The result of the analysis is “volume” for each rectangle/signal. Volume represents the sum of the intensity of all pixels in a rectangle corrected for the background.
5. The concentration of formed pyrophosphate is calculated as cP32PPi=IP32PPi/IP32ATP+IP32PPi×c0ATP, where *c* is concentration, *c*_0_ is initial concentration and *I* is the intensity (called a volume in imagequant) of a rectangle/signal.
6. For every reaction, pyrophosphate concentration *vs* time is plotted (Fig. 3A). Each time‐course is analysed by linear regression and the given slope represents the initial velocity. Finally, initial velocities are fitted against the amino acids (substrate) concentrations to generate the Michaelis–Menten fit by nonlinear regression from which the maximal velocity (*V*_M_) and Michaelis constant (*K*_M_) are extracted (Fig. 3B).

Using this assay, the activation reaction was followed for *Escherichia coli* IleRS, *E. coli* LeuRS, and *Priestia megaterium* IleRS2. Determined kinetic parameters are shown in Table 1, “label γ‐[^32^P]ATP” column. Comparison with previously published data [35, 36, 37] (Table 1, “label [^32^P]PP_i_^”^ column) reveals a good agreement, implying that the method we describe here ([^32^P]ATP/PP_i_ exchange) is a reliable substitution for the formerly used ATP/[^32^P]PP_i_ exchange assay.

**Table 1:** Kinetic parameters obtained for amino acid activation by EcIleRS, PmIleRS2, and EcLeuRS. The values represent the best fit value ± SEM of at least three independent experiments.

Enzyme + substrate	Label			
	γ‐[^32^P]ATP		[^32^P]PP_i_	
	*k*_cat_ (s^−1^)	*K*_m_ (μm)	*k*_cat_ (s^−1^)	*K*_m_ (μm)
EcIleRS + Ile	85 ± 6	5 ± 1	56.7 ± 0.3^a^	3.4 ± 0.1^a^
EcLeuRS + Leu	72 ± 10	40 ± 3	66 ± 2^b^	50 ± 2^b^
PmIleRS2 + Ile	50 ± 2	65 ± 7	66 ± 2^c^	49 ± 2^c^

^a^ Parameters reported in [35].

^b^ Parameters reported in [36].

^c^ Parameters reported in [37].

## Tips & Tricks

Note 1: When dealing with poor amino acid substrates, which need to be added in high concentrations, one should be wary of pH changes. We typically check the pH of a reaction mixture with the highest amino acid concentration using a pH strip. If needed, a buffer can be added in higher concentration and, preferably the pH of the amino acid stock solution should be adjusted.

Note 2: If *K*_M_ for the ATP is high the reaction mixture can be supplemented γ‐[^32^P]ATP activity higher than 0.0001–0.001 mCi·mL^−1^. We tested reactions with ATP up to 15 mm and have not observed a decrease in the assay sensitivity.

Note 3: One should try its best to freeze/thaw the ATP as few times as possible to prevent spontaneous hydrolysis.

Note 4: When longer reactions are followed, quenched time points can vaporize. To prevent this, time points can be quenched on a different plate.

Note 5: The polyethyleneimine layer of TLC plates can be unevenly distributed which can lead to poor product separation. To overcome this, time points can be spotted wider than 1 cm.

Note 6: Due to the high urea content in the TLC mobile phase, crystallization can occur easily, so it is best to use a mobile phase as fresh as possible.

Note 7: If, for any reason imaging fails, the erased phosphor storage screen can be re‐exposed to a TLC plate.

Note 8: When dealing with low activities it is best to use a freshly erased phosphor storage screen. Background caused by cosmic radiation accumulates over time when screens are not used.

Note 9: If signals are poorly developed or extremely irregularly shaped one can draw the polygon area in imagequant.

Note 10: When using multiple TLC plates, it is best to use multiple background corrections as the plates themselves can cause different background signals.

## Conflict of interest

The authors declare no conflict of interest.

### Peer review

The peer review history for this article is available at https://www.webofscience.com/api/gateway/wos/peer‐review/10.1002/2211‐5463.13903.

## Author contributions

IG‐S conceived the research idea; IZ, MD, and PK performed research; IZ, MD, MM, and IG‐S participated in the research development; IZ wrote the first draft; IZ and IG‐S wrote the final manuscript.

## Data Availability

The data are available from the corresponding author upon reasonable request.
